# Neuropsin-expressing cells in the retina affect melanopsin expression and response to light in mice

**DOI:** 10.1038/s41598-025-22815-4

**Published:** 2026-01-08

**Authors:** Hugo Calligaro, Brian Khov, Keun-Young Kim, Hiep Le, Mark Ellisman, Satchidananda Panda

**Affiliations:** 1 Salk Institute for Biological Studies, La Jolla, CA 92037 USA; 2 Department of Neurosciences, University of California at San Diego School of Medicine, La Jolla, CA 92161 USA; 3 National Center for Microscopy and Imaging Research, University of California, La Jolla, San Diego, CA 92161 USA

**Keywords:** Retina, Scanning electron microscopy

## Abstract

Neuropsin (OPN5) is an opsin involved in several non-image forming functions in the retina, such as the photoentrainment of the retinal circadian clock. These roles were previously attributed to melanopsin (OPN4) which prompted the hypothesis that OPN4 and OPN5 functionally interact. However, the specific cells expressing OPN5 and their interactions with the broader photoreception system remain unclear. This study aims to characterize OPN5-expressing cells and assess how their absence influences the photoresponses of OPN4-expressing cells. Through the use of genetically encoded reporters, confocal microscopy and serial blockface electron microscopy, we identified and reconstructed OPN5-cells and their processes. OPN5 expression was found in a small number of various retinal ganglion cell (RGC) subtypes, without overlap with OPN4-expressing cells, and presented distinct dendritic arborization patterns. In parallel, we measured the retinal electrical response to light in mice lacking either OPN4 or OPN5 to determine the contribution of each opsin and the consequences of their absence. Mice deficient in OPN5 exhibited reduced maximal discharge rates upon stimulation of light, though response duration remained unchanged. No specific electrical response to UV light was detected in OPN5-expressing cells suggesting that the response of OPN5 cells to light is translated in non-electrical pathway.

**Supplementary Information:**

The online version contains supplementary material available at 10.1038/s41598-025-22815-4.

## Introduction

 Adaptation to ambient light is of critical importance to the majority of living creatures on earth. In mammals, retina is a nervous tissue lining the back of the eye and is considered the only known photosensitive tissue allowing the integration and transfer of this light information to the brain. Retina is composed of cells specialized in receiving and processing of light information, organized into three cellular layers, the photoreceptor layer (PLR), the inner nuclear layer (INL), and the ganglion cell layer (GCL), and two plexiform layers, the outer and inner plexiform layers (respectively, OPL and IPL). The retinal ganglion cells (RGC) present in the eponymous layer are the last relay between the retina and the brain. To date, more than 40 subtypes of RGC have been identified using morphology, gene expression, and response to light^1,2^. One fundamental morphological feature for RGC classification is their dendritic arborization into the ON and OFF sublayers of the IPL. The names of those sublayers originate from the electrical response of RGC to light. RGCs with dendrites projecting into the ON IPL sublayer increase their discharge rate during a light stimulation, while the same stimulation decreases or abolishes the activity of some other RGCs with dendrites projecting into the OFF IPL sublayer. Some RGCs have dendrites in both the ON and OFF sublayers of the IPL and show increased activity when the light stimulation starts and ends.

In addition to the classical photopigments in rods and cones to support image-forming vision, mammalian retina contains another photopigment, melanopsin (OPN4), expressed in a subset of RGC, named melanopsin RGC (mRGC). These cells are directly photosensitive and have a pivotal role in regulating non-image-forming functions. They are considered to be the only cells directly projecting to the suprachiasmatic nucleus (SCN), the central circadian clock in the ventral hypothalamus. mRGCs were progressively characterized and classified into 6 subtypes (M1 to M6) based on different criteria including the level of melanopsin expression, the morphology (size of the soma and dendrites arborization), the expression of specific markers/genes, and intrinsic response to light^3^.

Another photopigment present in the mammalian retina, named neuropsin (OPN5), was identified two decades ago but only recently gained more attention as it started being associated with various physiological functions (reviewed in^4^). Specifically, the expression of OPN5 in the mammalian retina was initially linked to the light entrainment of the retinal circadian clock^5^ and was then proposed to regulate the post-natal regression of blood vessels in the retina^6^. In addition, mice lacking OPN5 also lost the ability to be entrained by low-intensity UV light^7^. These functions were until then very similar to the functions of OPN4 regulates; gestational eye development^8^, supports photoentrainment of the SCN^9,10^, and, in the absence of OPN4, the rhythmic expression of clock genes in the retina is disturbed^11^.

Recent studies have shown overlapping expression of OPN4 and OPN5 in RGCs. A study aimed at identifying the subtypes of OPN5 expressing cells in the retina using light-response properties^29^, identified many RGCs with OPN5 expression. Although the main subtypes they identified were F-mini-ON and HD2 subtypes, there was a small proportion of three mRGCs subtypes, M2, M5 and M6. None of the OPN5-expressing mRGCs was found to send projection to the SCN and IGL, which are densely innervated by other mRGC subtypes.

In the present study, we wanted to characterize the subpopulation of RGCs expressing OPN5 in adult mice by using morphological criteria and a combination of light and electron microscopy. We additionally attempt to identify OPN5-specific light response and the impact of OPN5 absence on the OPN4 response to light in the retina.

## Results

### Characterization of neuropsin-expressing cells in the retina in light microscopy

**Fig. 1:**
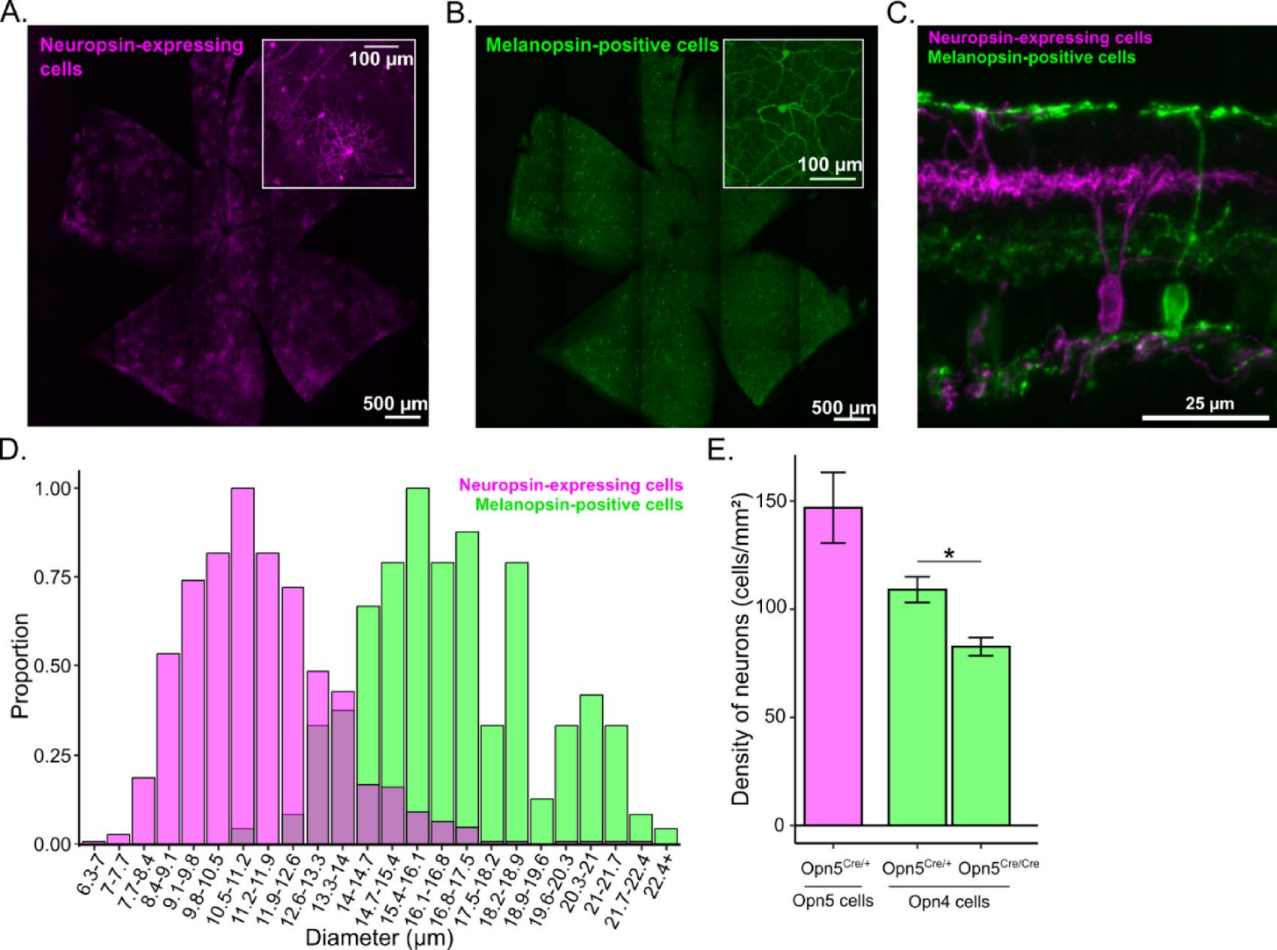
Expression of neuropsin and melanopsin in the retina. (**A**,**B**) Representative example of neuropsin (**A**) and melanopsin (**B**) expression in a wholemount mouse retina (scale bar = 500 μm). The insert shows a zoom on the peripheral retina (scale bar = 100 μm). (right) Schematic representation of all neurons expressing neuropsin in the retina. (**C**) Vertical section of a retina with neuropsin (red) and melanopsin (green) cells labelled (scale bar = 25 μm). (**D**) Distribution of the soma size of neuropsin (magenta) and melanopsin (green) expressing cells in retina (*n* = 1184, from 3 retina for each genotype). (**E**) Quantification of OPN5 cells (magenta, *n* = 4) and OPN4 cells (green) in OPN5^Cre/+^ (*n* = 5) and OPN5^Cre/Cre^ (*n* = 3) mice. Mann-Whitney U Test; *= *p* < 0.05.

In our efforts to characterize retinal cells expressing neuropsin, we employed the *OPN5-Cre* strain^6^. Through intravitreal injection of AAV carrying a Cre-dependent fluorescent reporter, we achieved the specific labeling of cells expressing neuropsin (OPN5, Fig. 1A) and immunolabelled melanopsin expressing cells (OPN4, Fig. 1B).

OPN5-expressing cells were found distributed throughout the entire retina, with no discernible specific pattern of distribution (as depicted in Fig. 1A). Notably, many of the labeled neurons exhibit dendritic and neuronal features, strongly indicating their identity as retinal ganglion cells (RGCs). This characterization was further confirmed through examination of retinal cryosections (as shown in Fig. 1C). Importantly, all neurons labeled by the fluorescent reporter were RGCs on all sections imaged. We only observed one occurrence of a labelled cell outside of the GCL. This cell was observed in the INL, possibly a displaced RGC. In addition, we did not observe any cell stained for both neuropsin and melanopsin. We measured soma sized of melanopsin- and neuropsin-expressing cells and established that their soma diameter distribution greatly varies (Fig. 1D). In average, melanopsin-expressing cells have larger soma than neuropsin-expressing cells (melanopsin: 26.16 ± 0.19 μm; neuropsin: 10.65 ± 0.06 μm). We counted a density of 146.9 ± 16.3 OPN5-positive cells per mm^2^ with AAV labeling in *OPN5*^*Cre/+*^ retinas (Fig. 1E).

We immunolabeled OPN4 in *OPN5*^*Cre/+*^ and *OPN5*^*Cre/Cre*^ retinas to compare the density of OPN5-positive neurons with mRGCs and assess the impact of the absence of OPN5 on mRGC population. Indeed, in OPN5-Cre strain, the Cre-recombinase was inserted in place of *Opn5* exon 1, erasing the expression of OPN5 protein^6^. *OPN5*^*Cre/Cre*^ are thus deficient in OPN5. We observed a significatively lower number of OPN4-positive neurons in these *OPN5*^*Cre/Cre*^ retinas (*OPN5*^*Cre/+*^: 109.09 ± 5.93 mRGCs/mm^2^, *OPN5*^*Cre/Cre*^: 82.72 ± 4.22 mRGCs/mm^2^; *p* = 0.036; Fig. 1E). To note, it has been shown that immunostaining of OPN4 only labels mRGCs subtypes with the highest expression of OPN4, M1-M3 ^3^. It is, however, unclear if the absence of neuropsin directly impacts mRGCs intrinsic light response, melanopsin expression, or if it is a consequence of retinal vasculature overgrow previously identified in absence of neuropsin^6^. Overgrowth of blood vessels can cause increased leakage and leads to defect of visual functions^13,14^.

### Brain projection of OPN5 expressing cells

**Fig. 2:**
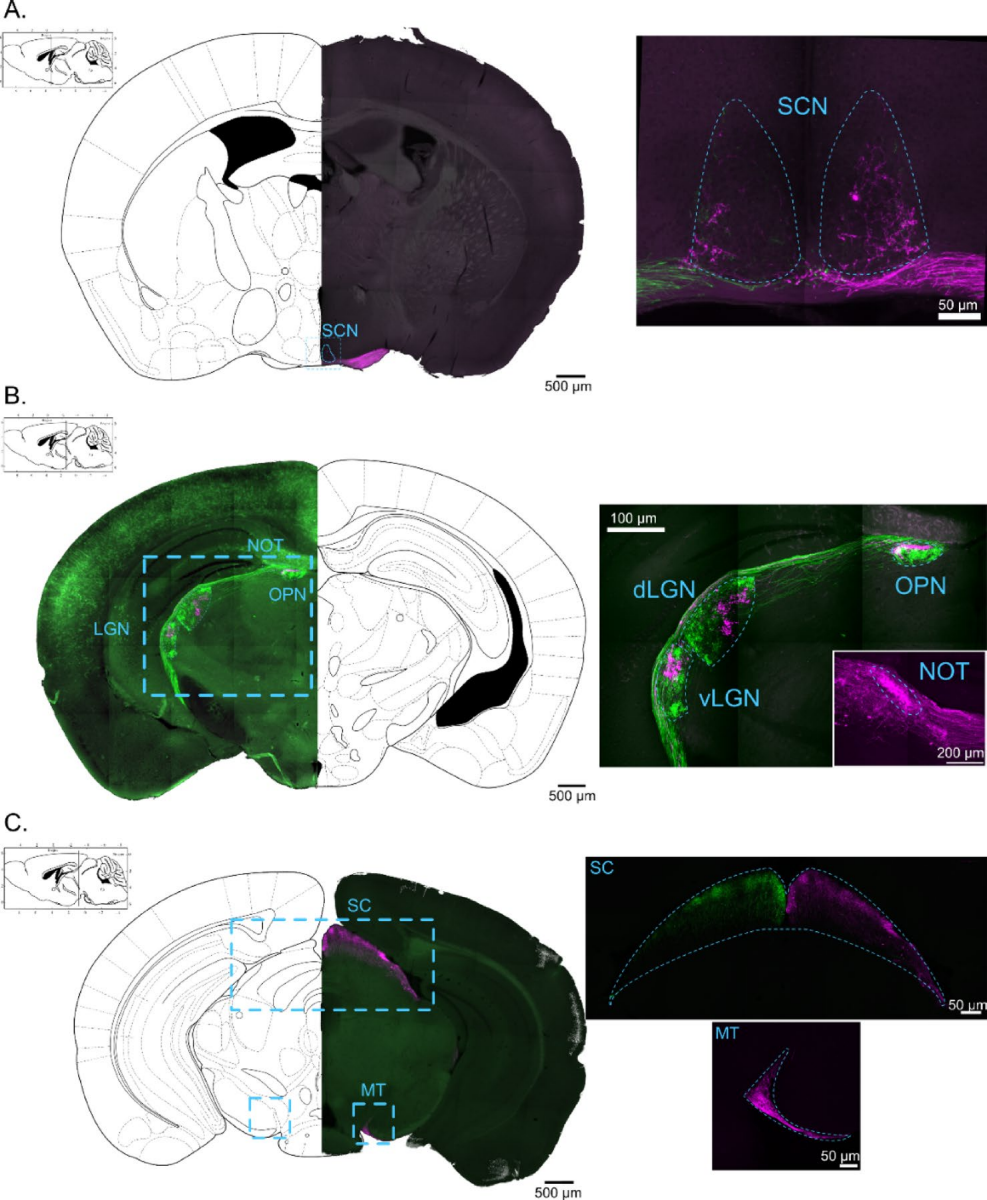
Projection of neuropsin expressing retinal ganglion cells to the brain. Neuropsin RGCs weakly project to the suprachiasmatic nucleus (SCN) (**A**), have strong bilateral projections to the lateral geniculate nucleus (vLGN, dLGN) and olivary pretectum nucleus (OPN), and unilateral projections to the nucleus of the optic tract (NOT) (**B**), to the superior colliculus, and to the accessory nuclei (**C**).

To identify the bilateral projections to the brain of OPN5-expressing cells, we injected AAV expressing Cre-dependent EGFP (left eye) and AAV expressing Cre-dependent tdTomato (right eye) in *OPN5*^*Cre/+*^ mice eyes. This approach extensively labels dendrites, soma as well as axonal processes in Cre-expressing neurons. Axons from OPN5-positive neurons exhibit diverse projections to multiple brain regions associated with visual functions. A few axonal branches were observed within both the core and shell regions of the hypothalamic SCN that serves as the master circadian oscillator (Fig. 2A). However, the OPN5 positive axons in the SCN are notably sparser than the mRGC axons^15^ that serve as primary conduit of light input to the SCN. Beyond the hypothalamus, OPN5 axons project to the ventral (vLGN) and dorsal (dLGN) lateral geniculate nucleus, with a clear segregation of axons of ipsi- and contralateral origin (Fig. 2B). The dLGN is a relay to the visual cortex and involved in the scene perception^16^ while vLGN has recently been describe to modulate response to threats^17^. Contralateral neuropsin cell axonal terminals are concentrated in the dorsal part of the dLGN, which has been described as innervated mainly by direction-sensitive RGCs^18^, and the external vLGN, the retinorecipient part of the vLGN^19^. The ipsilateral terminals in both vLGN and dLGN are localized in the central patches. No overlapping between ipsi- and contralateral terminals are observed in these structures. The intergenicular lamina (IGL), localized between vLGN and dLGN, appears to be spared by OPN5-expressing axons terminals. In the thalamic region, the olivary pretectum nucleus (OPN), implicated in pupil constriction, on the other hand, shows substantial overlap of bilateral axons from OPN5-expressing cells (Fig. 2B). Although the OPN harbors synaptic terminals from RGCs, including mRGCs^15^, the mRGC axonal processes are known to be more numerous than other RGCs in the OPN. Ipsilateral terminals are concentrated in the shell of the OPN while contralateral terminals are mainly in the core of the OPN, with some overlap in the lateral part of the shell. Finally, the axons of neuropsin-expressing cells project to the nucleus of the optic tract (NOT), the superior colliculus (SC) and the accessory nuclei (MT) (Fig. 2B, C). These regions are involved in gaze-stabilization and in image forming. These axons predominantly display a contralateral pattern of innervation within these structures. This pattern of projection further underscores the potential involvement of neuropsin-expressing cells in visual processing (Fig. 2C). In summary, the results suggest a distinctive role for OPN5-expressing cells in these neural circuits and potentially highlights their unique contributions to visual and circadian processes.

### Reconstruction of neuropsin-expressing RGCs using serial blockface scanning electron microscopy

Characterizing RGC subtypes based solely on morphological parameters poses a considerable challenge and demands a level of resolution adequate to distinguish the dendritic arborizations of each RGC subtype. This task becomes particularly difficult with OPN5-*e*xpressing RGCs, as observed in light microscopy because their dendrites largely overlap (Fig. 1A). The limitations of light microscopy highlight the necessity for electron microscopy, which not only provides the essential resolution but also grants access to the intracellular content of examined cells, including mitochondrial populations and synaptic sites.

**Fig. 3:**
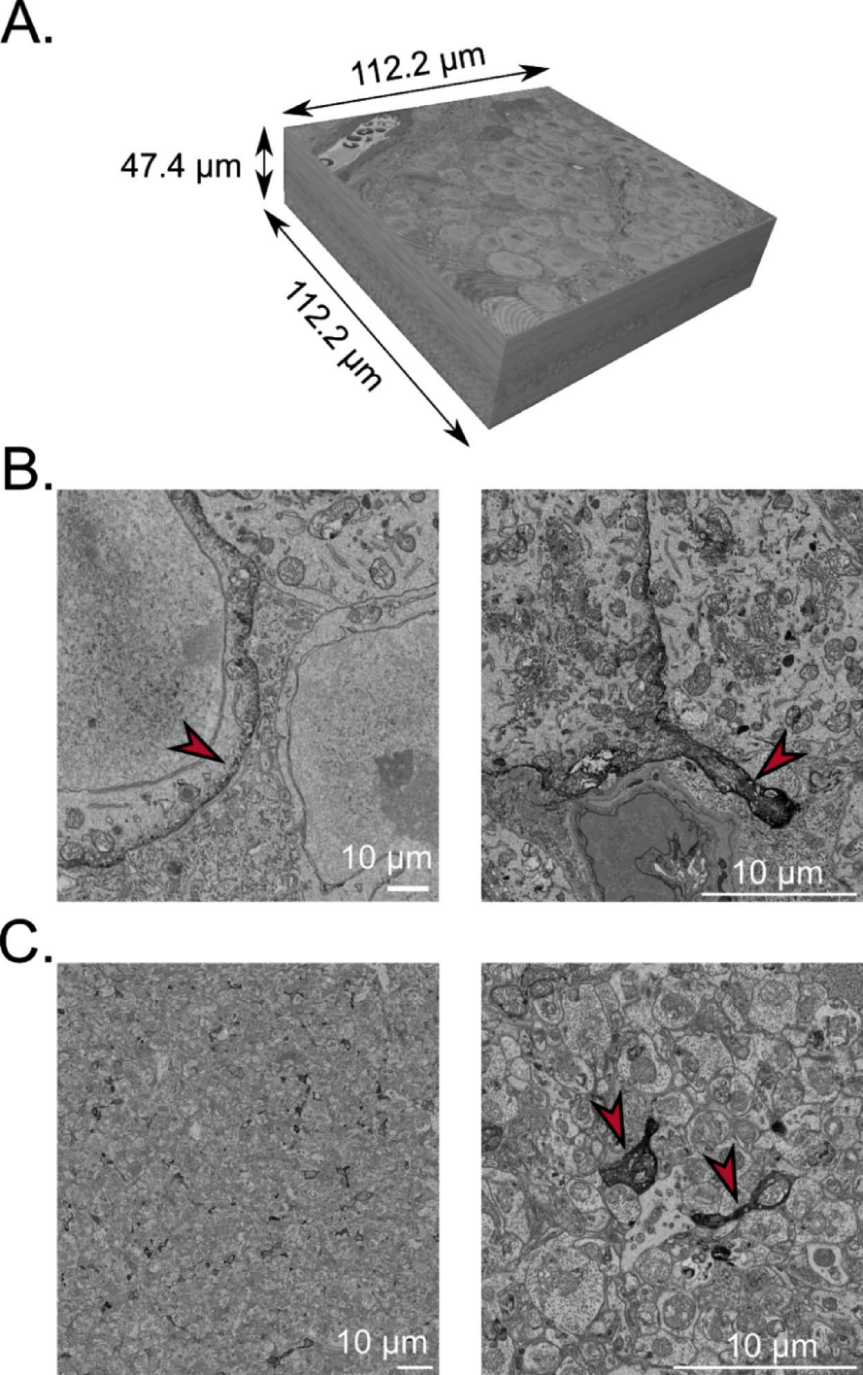
Serial blockface electron microscopy image volume of the retina with APEX2-labelling of the neuropsin-expressing cells. (**A**) Size of the volume generated, ranging from the ganglion cell layer to the inner nuclei layer. The image volume comprises 948 sections, each measuring 50 nm in thickness. Each section consists of an array of 20,000 by 20,000 pixels, achieving a high resolution of 5.61 pixels per nanometer. (**B**) Representative image of the APEX2 labelling on the membrane of neuropsin-expressing cells in the soma (left) and in the base of a dendrite (right). (**C**) APEX2-labelled dendrites in the inner plexiform layer at low (left) and high magnification (right). Scale bars = 10 μm.

Employing advanced techniques like serial blockface scanning electron microscopy (SBEM), coupled with specific EM staining for OPN5 cells, allowed us to reconstruct and identify these RGC subtypes. To produce EM-grade OPN5-specific staining of RGCs, we intra-vitreally injected 2 AAVs to express Cre-dependant EM reporter APEX2^20^ and Cre-dependant fluorescent marker tdTomato to a male *OPN5*^*Cre/+*^ mouse. After 4 weeks, the retina was fixed and processed to reveal APEX2 labeling. APEX2 and tdTomato signals were compared to verify the efficiency of the labeling. We then chose a region of the peripheral retina containing multiple stained RGCs somas to image in EM. The final SBEM 3D image volume consisted of 948 sections with each covering 113.2 μm x 113.9 μm area with inter-section interval of 50 nm. The volume contained a total of 90 neuronal soma on a surface of 12,893 μm^2^ (~ 7500 neurons/mm^2^). The image volume extended from the GCL to the inner limit of the INL, for a total volume of about 600,000 µm^3^ (Fig. 3A). A total of 4 neuropsin-expressing neurons were identified by their darkened cell membrane (Fig. 3B) marked by APEX2 labeling. Their neurites were also labeled and appeared almost completely dark (Fig. 3C), thus allowing us to easily follow the neurites through the sections. The dendrites regularly present swellings containing mitochondria and form synapses with nearby axons. The other organelles of the cells were not labeled by APEX2.

### Semi-automatic 3D reconstruction of OPN5-expressing cells

Our initial step involved the automated segmentation of APEX2-labeled dendrites. We achieved this segmentation by leveraging the visual distinction between these dendrites and other cells. Our method involved a straightforward pixel value detection technique, the details of which can be found in the Methods section. In brief, the threshold detection applied to the whole volume created 1,000,038 contours matching our pixel intensity and size thresholds. The IMOD functions *imodmesh* and *imodsortsurf* sorted these contours based on their interpolated 3D surfaces into 132,312 objects. Using a custom Python script, we removed all objects smaller than 15 sections to eliminate small false-positive elements such as lysosomes or mitochondria. The number of objects was reduced to 3000 that way. Finally, we manually checked the remaining objects to verify they are all dendrites and merged objects corresponding to the same cell (Supplementary Video 1). To evaluate the quality of the prediction, we manually check each object created and establish the false-positive rate (FPR) in the GCL and the IPL (Supplementary Fig. 1). The GCL had a relatively high FPR (71%), due to large structures dense to electrons which escape our filtering steps, such as lysosomes, nucleoli or Muller cells cytoplasm. In the IPL, the FPR was notably lower (41%); the structures falsely segmented were processes of Muller cells. This pipeline of semi-automatic segmentation allows a fast morphological characterization of cells with dark labelled neurites and can be easily included in connectomics studies workflow.

**Fig. 4:**
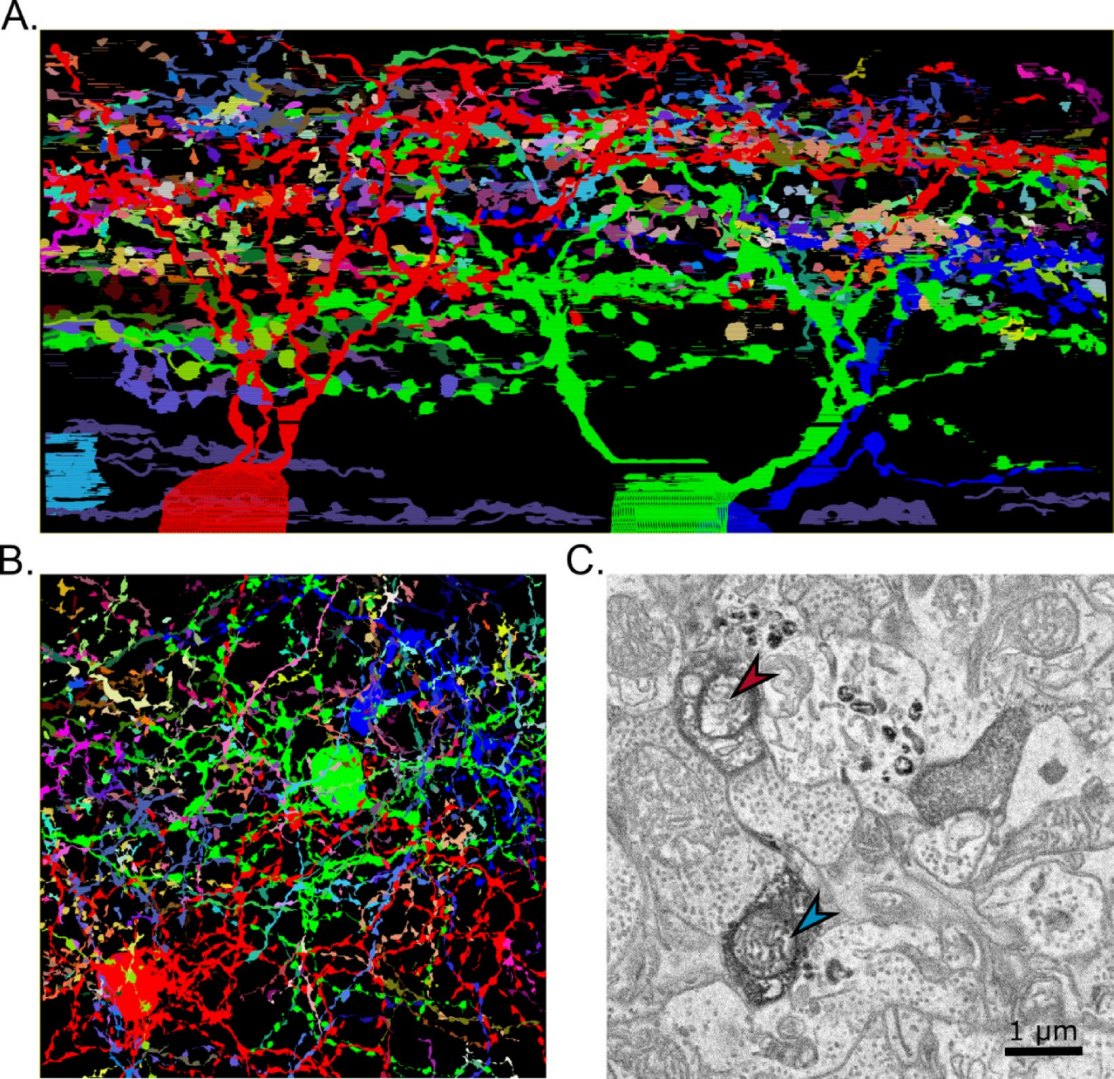
Autosegmentation of the SBEM volume. (**A**) Result of automatic segmentation using pixel value detection. The result was proofread to delete false-positive objects such as lysosomes. Four APEX2-labelled cells were detected (red, green, blue and light blue). The purple objects close to the soma are axon fibers. (**B**) Top view of the result of the automatic segmentation. (**C**) Example of dendrites with different level of APEX2 labelling. Blue arrowhead indicates a normally labelled dendrite, detected by the autosegmentation; red arrowhead indicates a weakly labelled dendrite that was not detected. Scale bar = 1 μm.

This segmentation process successfully identified the presence of four APEX2-labeled cells, with three of them exhibiting dendrites within the imaged volume. Additionally, a dense network of dendrites was observed throughout the entire inner plexiform layer (IPL) (Fig. 4A). These dendrites covered a large portion of the retina surface (Fig. 4B). While proofreading the segmentation, we however discovered that some dendrites were lightly labelled and, although clearly distinguishable from unlabeled neurites, were not automatically segmented (Fig. 4C).

**Fig. 5:**
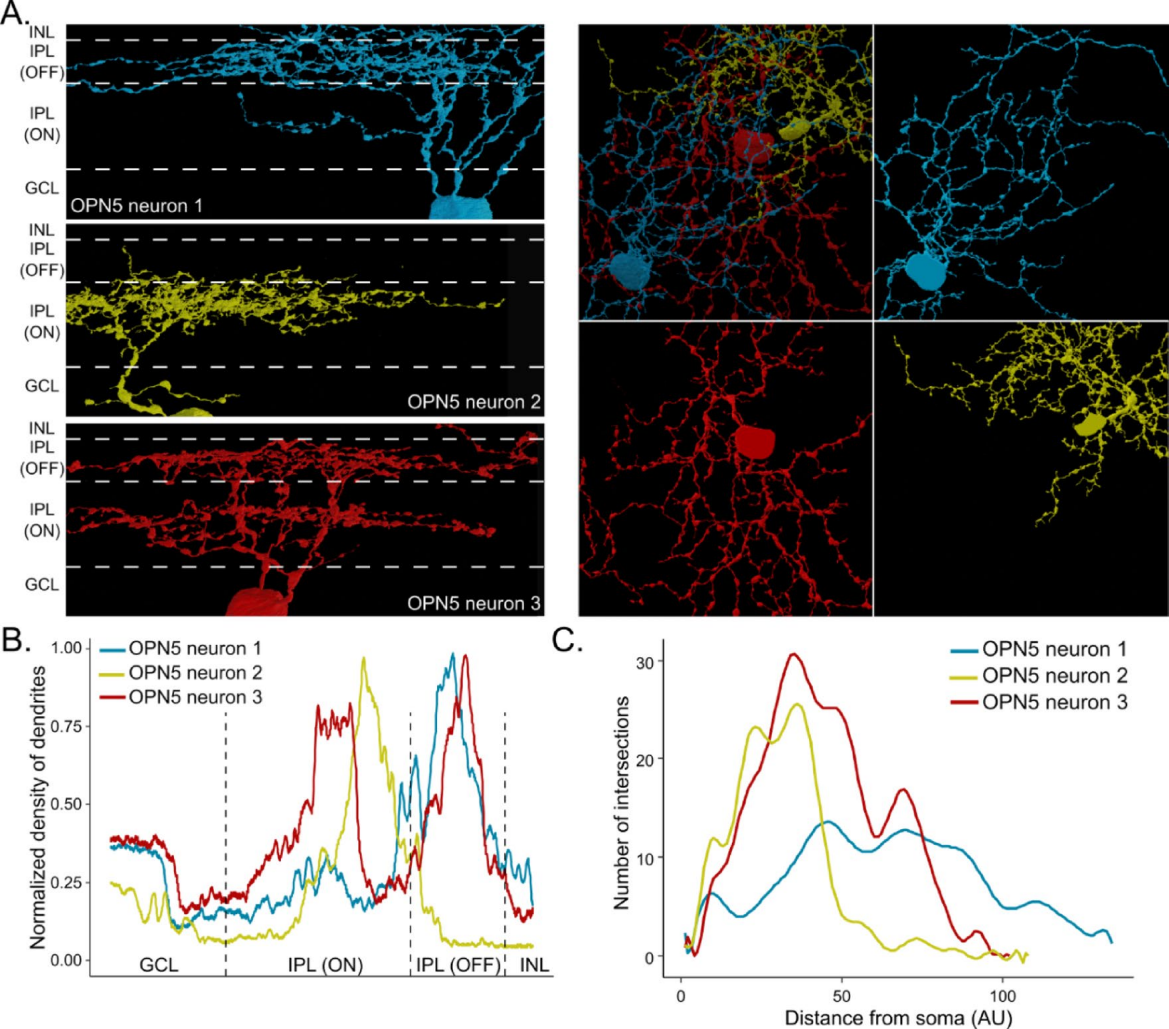
Fully reconstruct OPN5-expressing cells. (**A**) Side view (left) and top view (right) of the three fully reconstructed cells. (**B**) Quantification of the density of dendrites of each cell in the inner plexiform sublayers. (**C**) Sholl analysis of each cell in top view.

To ensure a comprehensive analysis of dendritic arborization for these cells and avoid the risk of missing any dendrites, we carried out a manual refinement of the autosegmentation process using VAST-lite software^21^. This involved systematically tracking and fully segmenting all APEX2 labeled dendrites until they either reached the volume’s edge or came to an end. As anticipated for retinal ganglion cells (RGCs), the dendrites exhibited distinct organization into specific sublayers of the inner plexiform layer (IPL). To our surprise, the dendrites of all three cells displayed unique branching patterns within the IPL. Specifically, OPN5 neuron 2 had dendrites predominantly terminating in the outer region of the ON sublayer of the IPL, while OPN5 neuron 1 exhibited termination in the OFF sublayer of the IPL. OPN5 neuron 3 had dendrites that extended into both the ON and OFF sublayers of the IPL. This distinctive segregation of dendritic arborization patterns suggests a specialization of these RGCs in processing specific types of visual information in the retina. (Fig. 5A). The distribution of dendrites within each sublayer of the retina was quantified to assess the relative density of dendrites (Fig. 5B). Some dendrites of all three neurons went out of the volume, making any measurement of their dendritic radius impossible. However, we were able to estimate the relative distribution of these dendrites in relation to their respective soma using a classical analysis for neurons morphology, the Sholl analysis^22,23^. Because of the dendrites continuing outside of the volume, the Sholl analysis would be only partial but the dendrites present in the volume were sufficient to establish the difference between the neurons. Indeed, we obtained very different profiles for the three neuropsin-expressing neurons. Neuron 2 has mostly branches close to the soma while neuron 1 dendrites spread farther away (Fig. 5C). We also quantified the content of each soma but a meaningful comparison is difficult as the volume starts from the middle of the soma. We thus estimated the portion of the visible soma occupied by the nucleus and mitochondria. All data are presented in Table 1.

**Table 1:** Morphological and cellular characteristics of neuropsin-expressing neurons.

	Soma diameter (µm)	Nuclei (% of soma)	Mitochondria (% of soma)	Mitochondria (#/µm^3^)	Dendritic arborization	Nb of initial dendrites
OPN5 neuron 1	15.24	22.78	8.81	0.81	OFF	3
OPN5 neuron 2	11.76	33.76	4.63	0.44	ON	1
OPN5 neuron 3	15.37	22.74	7.79	0.71	ON OFF	2

### Retina coverage and synaptic input of OPN5-expressing cells dendrites

Next, we calculated what fraction of the retina surface may be covered by OPN5 expressing cells. The dendrites of the three neuropsin-expressing cells collectively covered a substantial portion of the retinal surface, accounting for approximately 30.6% of the total retinal surface. However, it’s worth noting that within the imaged volume, there were numerous other dendrites expressing OPN5, which did not originate from these three cells. The initial result from the automated segmentation indicated that around 42% of this volume surface was covered by dendrites from OPN5-expressing neurons.

**Fig. 6:**
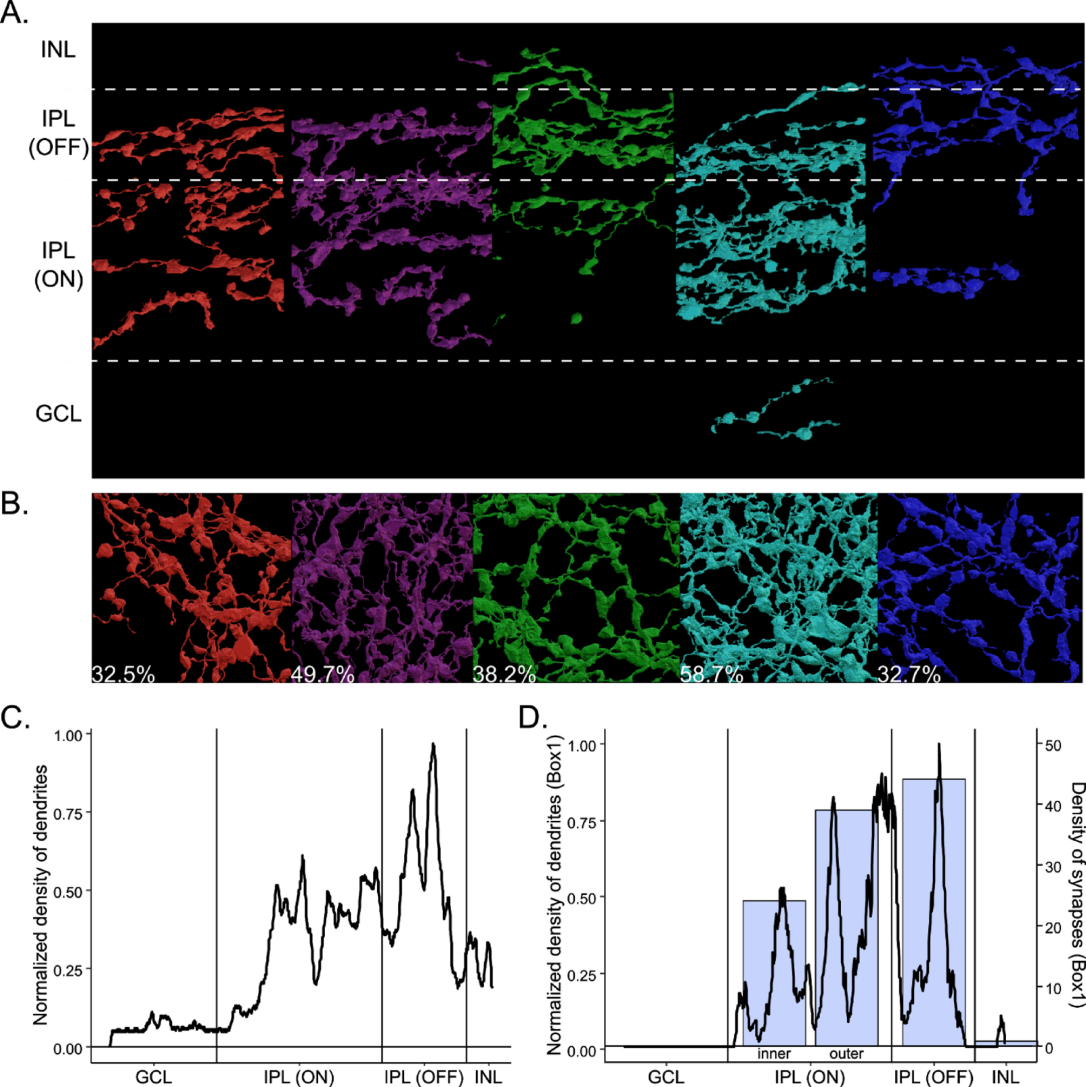
Retina coverage by OPN5-expressing dendrites. Side (**A**) and top (**B**) views of the 5 boxes with all OPN5-expressing dendrites segmented. (**C**) Quantification of the density of dendrites of all boxes in the inner plexiform sublayers. (**D**) Distribution of synapses density (blue boxes) on OPN5-expressing dendrites in the inner plexiform sublayers (black line) of Box1.

Recognizing that this percentage might be underestimated due to unsegmented dendrites, we adopted a strategy to obtain a more accurate representation. We partitioned the volume into 25 sections based on a 5 by 5 grid and randomly selected five of these sections for the full segmentation of all labeled dendrites. This approach allows us to gain a better understanding of the overall coverage and distribution of OPN5-expressing dendrites in the retina. (Fig. 6A). Dendrites were distributed in all layers of the IPL, with a preference for the OFF sublayer of the IPL and very few dendrites in the inner part of the ON sublayer, possibly proximal segments of dendrites from the somas of OPN5-expressing neurons (Fig. 6B). This distribution is consistent with the three cell subtypes we identified earlier, even though only a small portion of the dendrites traced in the boxes overlap with the dendrites from those 3 labeled OPN5-expressing neurons. Using this method, we confirmed that the OPN5-expressing dendrites covered an average of 42% of the retina surface in the current image volume, ranging from 32% to 58% (Fig. 6B).

We then assessed whether all layers of the retina contained OPN5-dendrites receiving synapses. We marked all synapses in one of the previously segmented boxes and compared the distribution of synapses to the distribution of dendrites across the sublayers of INL (Fig. 6C). We counted a total of 108 synapses distributed in proportion to the density of dendrites in the corresponding box (Fig. 6D). The inner ON sublayer had the lowest number of dendrites and synapses, while the OFF sub-layer had the highest density of dendrites and synapses. Thus, OPN5-cell dendrites receive synaptic inputs from all sublayers of the INL.

### Electrical response to light of OPN5- and OPN4-expressing cells

**Fig. 7:**
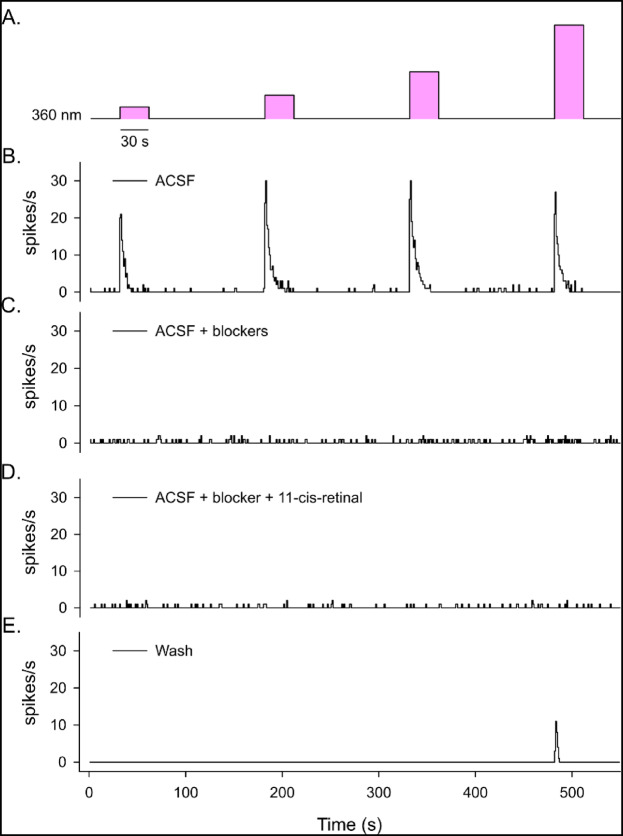
Example of MEA recording of OPN4^Cre/Cre^ mouse retina. (**A**) Light protocol used: Four 30 s UV-light (360 nm) stimulation of increasing irradiance. (**B**–**E**) Representative examples of the response to the light stimulations of one RGC perfused with aCSF (**B**), aCSF with synaptic blockers (L-AP4, D-AP5, CNQX) (**C**), aCSF with synaptic blockers and chromophore (11-cis-retinal) (**D**), and after 30 min of wash with aCSF only (**E**).

OPN5 is presumed to be a UV-sensitive opsin^26^. To verify if UV light stimulation of mouse retina can lead to an electrical response of neuropsin-expressing cells, we recorded light responses of the ganglion cell layer of the retinas of *OPN4*^*Cre/Cre*^ mice using multi electrode array (MEA). As these retinas lack melanopsin, the observed photoresponses should originate from the outer retina photoreceptors or from OPN5 neurons. We first recorded the retina piece perfused with aCSF only and applied four UV light stimulation of increasing irradiance (365 nm, from 1.17 10^11^ to 1.02 10^14^ photons/cm^2^/s) (Fig. 7A). Around 40% of the active cells showed a dose-depending response (Fig. 7B). To eliminate the response due to SW-cones, we then incubated the retinas in a cocktail of synaptic blockers for one hour. The same light protocol was then applied again to the retina. With the synaptic blockers, no response could be detected (Fig. 7C). A previous study showed that neuropsin cells might depend on the exogenous supply of retinal to respond to light^24^, we, therefore, incubated the retinas with the cocktail of synaptic blockers and 11-cis-retinal for 1 h before applying the same light protocol again. No light response was detected either (Fig. 7D). Finally, to verify that the absence of response was not due to a survival problem of the retina, retinas were washed with fresh aCSF, without synaptic blockers. After 30 min of washing, partial restoration of the light response was detected upon application of the light stimulations (Fig. 7E). Six retina pieces from 3 different OPN4^Cre/Cre^ mice were recorded and exposed to the same light protocol with the same absence of response in the presence of synaptic blockers. This suggests that OPN5-expressing cells do not have an electrical response to light in these conditions.

**Fig. 8:**
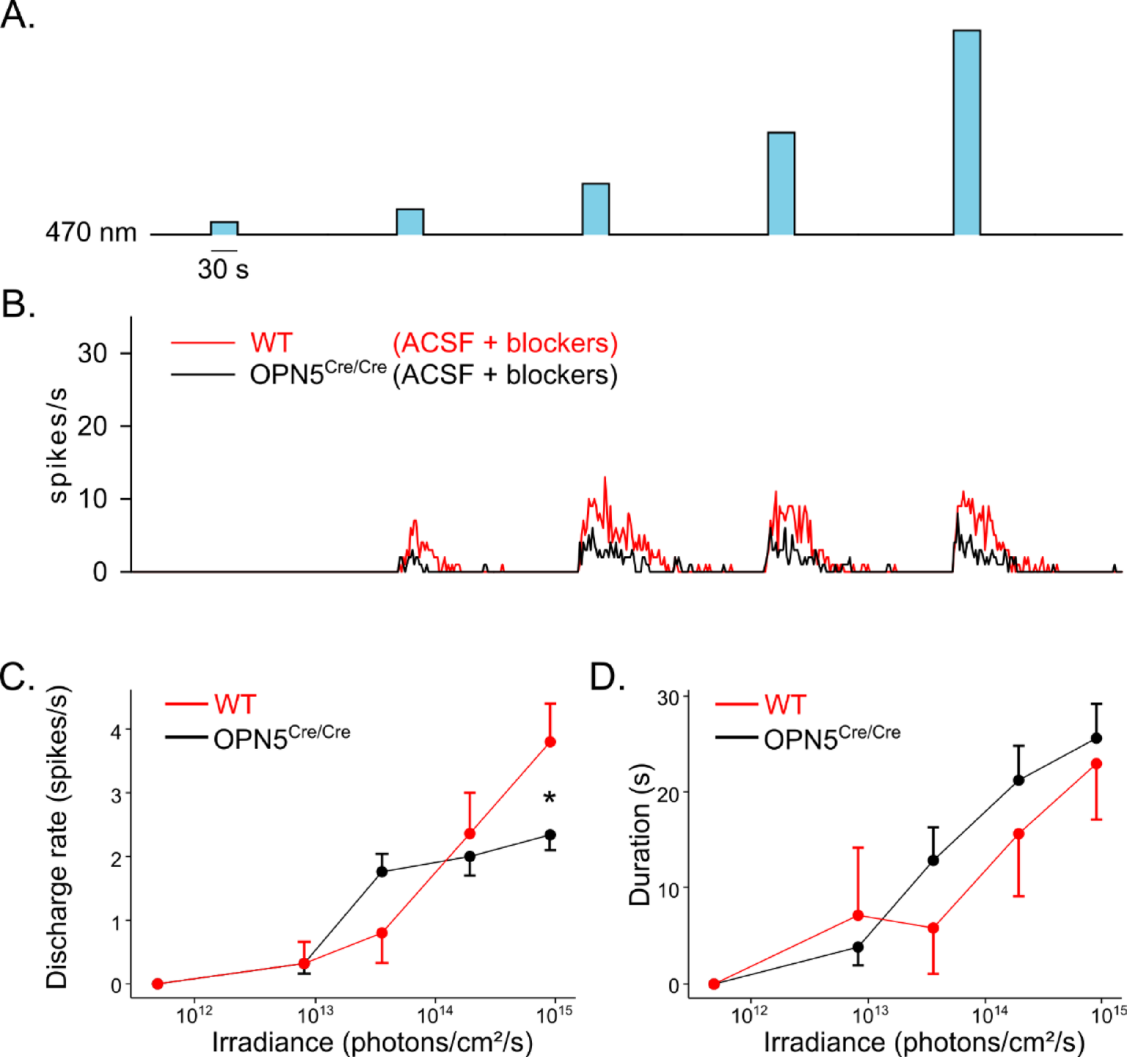
Absence of neuropsin reduce light response of melanopsin cells at high irradiances. (**A**) Light stimulation protocol applied; five 30 s stimulations of increasing irradiance (470 nm). (**B**) Representative example of the electrical response of mRGCs to the light stimulation in presence of synaptic blockers (L-AP4, D-AP5, CNQX). C, D. Discharge rate (**C**) and duration (**D**) of the response to light stimulation in WT (red) and OPN5 deficient mice (black). Pair-wise Mann-Whitney U test; * = p-value < 0.05.

We subsequently evaluated if the absence of OPN5 would also impact the light response of mRGCs. We recorded *OPN5*^*Cre/Cre*^ mouse retinas, which are deficient in neuropsin, on a MEA and exposed them to increasing intensity light stimulations (15 s, from 4.82 10^11^ to 9.01 10^15^ photons/cm^2^/s, Fig. 8A). We stimulated with 470 nm monochromatic light, close to the peak of the sensitivity of OPN4^25^ and used five stimulations to show a better resolution of the response. We obtained the expected intensity-dependent response from mRGCs from both WT and *OPN5*^*Cre/Cre*^ retinas (Fig. 8B). We however observed a reduced average discharge rate in *OPN5*^*Cre/Cre*^ at the highest irradiance (WT: 3.91 ± 0.60 spikes/s, *n* = 107; *OPN5*^*Cre/Cre*^: 2.35 ± 0.25 spikes/s, *n* = 19; *p* < 0.05; Fig. 8C) without any significant change to the total duration of the response (WT: 22.90 ± 5.86 s; *OPN5*^*Cre/Cre*^: 25.63 ± 3.54 s; *p* = 0.67; Fig. 8D).

## Discussion

Neuropsin-expressing and melanopsin-expressing neurons in the retina share many similarities and some differences. They are both non-visual opsins expressed in subpopulations of RGCs and regulate both circadian-related and non-circadian functions. However, we found that neuropsin neurons do not show specific electrical responses to light, whether UV or visible, and probably rely on a different mechanism to respond to light. Kojima and colleagues (2019) showed that UV-activation of OPN5 expressed in cell line induce a rapid increase of G_i_-type G protein and a reduction of cyclic adenosine monophosphate (cAMP), an intracellular second messenger^26^. This observation also support a decrease of c-Fos expression in response to UV-light stimulation in RGC of a mice lacking key element for rods, cones, and melanopsin photoreception^27^. Taken together, these results suggest that OPN5-expressing cells respond to UV-light via an cAMP-associated intracellular molecular cascade that still need to be fully characterized.

In contrast, melanopsin-expressing cells respond to light within seconds of light stimulation. Previous studies showed that in a model of mice lacking all photoreceptors but neuropsin, shifting the retinal clock requires particularly long and intense light stimulation (3 h, 10^15^ photons/cm^2^/s)^5,28^, which could be due to the absence of direct electrical response to light of OPN5-expressing cells. In contrast, rod photoreceptors require much shorter and dimmer light pulses (30 min, 10^14^ photons/cm^2^/s) to achieve the same result^12^. Mice deficient in neuropsin also show impairments of their locomotor activity entrainment under very dim UV light^7^. However, the precise role of neuropsin in entraining locomotor activity is unclear, as the main brain regions regulating this function (SCN, IGL) do not receive direct photic information from neuropsin-expressing cells. Although we observed a few neuropsin-expressing cell axons in the SCN, it is unlikely that they are sufficient for light entrainment. The SCN and IGL are known to be almost exclusively innervated by mRGCs^15^.

Two studies counted the population of OPN5 using different labelling approaches and obtained a density of cells at least two time superior (320–520 cells/mm^2^^5,29^). These studies used, respectively, 8 days old Opn5^−/−^ mice carrying a Knock-In tau-lacZ gene under the control of OPN5 promoter and adult OPN5-Cre mice bred with transgenic mice carrying a Cre-dependant fluorescent reporter (Ai14). The same group evaluated the density of OPN5-expressing cells in adult OPN5-Cre mice after intraocular injection of AAV expressing Cre-dependent tdTomato, and observed up to 85% of co-expression of Ai14 and tdTomato. Our experiment used a similar viral approach to label OPN5-expressing cells in the same OPN5-Cre strain but we observed a sparser population of labeled cells. This could be explained by a lower infectious efficiency of our intra-vitreous injections. This could also explain the absence of spatial distribution observed in our study while D’Souza and colleagues (2022) observed a higher number of OPN5-expressing RGCs in the dorsotemporal quadrant and a reduced density in the ventrotemporal quadrant^29^. This possible lower efficiency do not however impact the specificity of the viral approach, which is well documented for the OPN4-Cre strain^30,31^. Furthermore, the projections to the brain of OPN5-expressing RGCs that we described here match the observations done previously^29^, with the notable exception of the dorsal terminal nucleus that did not receive axon terminals in our experiments.

Our reconstruction of OPN5-expressing cells confirmed here the conclusion obtained by another group using a functional approach^29^: in the mouse retina, OPN5 does not appear to be expressed in a particular subtype of RGC but is expressed in multiple subtypes of cells. Using an online reference database (rgctype.org, Northwestern University, Chicago), we established that the three reconstructed cells have morphological similarities with F-mini-ON, HD1 and ON-OFF-direction sensitive RGCs. These RGCs subtypes were also identified by D’Souza and colleagues (2021) using a functional approach^29^. Furthermore, the distribution density of OPN5 expressing RGCs dendrites within the IPL observed in electron microscopy corresponds to the distribution observed previously in fluorescence microscopy^29^.

Our SBEM dataset also revealed dendrites with weaker APEX2 labeling, which are not present in the fully reconstructed cells. This weak labeling cannot be attributed to technical issues, as dendrites with varying labeling strengths are found adjacent to each other. The distance between the dendrite and the cell soma is also unlikely to be the cause, as weakly labeled dendrites were observed in all parts of the IPL. It is possible that a weaker OPN5 expression by the RGC could be responsible for this weaker labeling, although a full cell reconstruction with weak labeling is necessary to confirm this hypothesis. A similar expression variation was also seen in different subtypes of mRGCs^3^.

More interestingly, we observed a reduced expression and lower electrical response to light of melanopsin in retina deficient in neuropsin. This is particularly puzzling as melanopsin and neuropsin are expressed in separate sets of RGCs in adults. Given the previous report on the effect of the absence of neuropsin on the local entrainment of the retinal clock^5^, one explanation could be that the difference observed would be due to a circadian misalignment between WT and OPN5 deficient retinas. The circadian misalignment could lead to a difference in melanopsin expression and light sensitivity. However, previous experiments have established that the retinal clock entrains to white light with no UV-component in vitro, either by examining clock gene expression^28^ or by measuring one of the clock outputs, melatonin^32^. Similarly, PER2 rhythms in the retina of mice deficient in neuropsin were in phase with WT retina not exposed to any light cycle in vitro^5^. The retinal clock might be entrained by rod photoreceptors^12^ or other factors in vivo. Thus, circadian misalignment is unlikely to cause the observed differences between WT and OPN5 deficient retinas. Another possible explanation could involve the developmental period of the mRGCs as D’Souza and colleagues showed that a fraction of mRGCs express OPN5 during early developmental stage^29^. Additionally, Nguyen and colleagues (2019) have shown a reduction in dopamine level during post-natal development in OPN5 deficient mice retina^6^. Dopamine has been associated with the daily regulation of melanopsin mRNA in rats retina^33^, this reduction of dopamine thus could have lead to a reduction of melanopsin level.

It is currently unclear if these modifications of mRGCs response to light could have functional implications or participate in the phenotypes attributed to OPN5 deficiency. Future work should investigate if the absence of OPN5 also impacts the light response of classical RGCs, in particular RGCs that have been shown to express OPN5.

## Methods

### Animals

All animal care and procedures were approved by the Institutional Animal Care and Use Committee (IACUC) of the Salk Institute for Biological Studies and in accordance to ARRIVE guidelines. All the methods were carried out in accordance with relevant guidelines and regulations. A mouse strain expressing Cre-recombinase under the control of Opn5 promoter (*Opn5*^*Cre*^) was obtained from Dr. Richard Lang from Cincinnati Children’s Hospital Medical Center^6^. The OPN4-Cre and OPN5-Cre strain were maintained using heterozygotes animals as breeders. All experiments comparing genotypes were done with littermates. For histology and SBEM, male and female Opn5^Cre/+^ of 6–12 weeks of age were used. For electrophysiological recording, homozygotes OPN4^Cre/Cre^ and OPN5^Cre/Cre^ mice of 8 and 12 weeks of age were used. All mice were housed under a standard 12:12 light/dark cycle (~ 100 lx at cage level) in a temperature-controlled room (~ 22 °C). Food and water were available *ad libitum*.

### Viral vector

To label neuropsin cells with the fluorescent proteins EGFP and tdTomato, AAV2-EF1a-DIO-EGFPf and AAV2-EF1a-DIO-tdTomato were injected into, respectively, the left and right eye of Opn5^Cre/+^ mice. To label neuropsin-expressing cells for electron microscopy, we produced a vector with a farnesyl sequence cloned into the 3’ end of the APEX2 construct^20^ and inserted it in an inverted orientation between the lox sites in an AAV2-DIO vector^34^. This viral vector, AAV2-EF1a-DIO-APEX2-farnesyl, was co-injected with AAV2-EF1a-DIO-tdTomato into both eyes of Opn5^Cre/+^ mice to fluorescently label APEX2 expressing cells with tdTomato, so that these cells could be identified under a fluorescent microscope. All viral vectors were produced by the Salk Gene Transfer, Targeting and Therapeutics Viral Vector Core Facility at titers of 2.68 × 10^11^ GC/mL (APEX2-f), 9.43 × 10^11^ GC/mL (tdTomato), and 2.12 × 10^12^ GC/mL (EGFP-f).

### Vector injection

Anesthesia was induced with isoflurane (4%) and maintained (2%) until the end of the procedure. The mouse was placed on one side under a dissection microscope so one eye was completely visible. Gentle pressure was applied around the eye so the edge of the sclera was visible. A small incision was made with a 27-gauge insulin needle 0.5 mm posterior to the *Ora Serrata*. The viral vector was loaded into a Hamilton microliter syringe with a 34-gauge beveled needle mounted on a micromanipulator. The micromanipulator was used to insert the loaded needle through the incision. The vector was slowly injected and allowed to diffuse through the vitreous humor for 2 min. The whole procedure was then repeated on the other eye with the appropriate vector. Upon delivery of the viral vector to both eyes, the animal was removed from isoflurane anesthesia and lubricant eye ointment (AKORN) was applied to both eyes. The animal was placed in a clean cage to recover and was returned to its home cage after 1–2 min when righting reflex was restored.

### Histology

#### Brain sample preparation

The mice were anesthetized using a xylazine/ketamine (ketamine: 100 mg/kg, xylazine: 10 mg/kg) solution via intraperitoneal injection, at ZT5-6. Once anesthetized, an incision was made below the ribcage and continued diagonally upwards to expose the heart. A 25-gauge needle was gently inserted in the left ventricle of the heart and a small cut was made in the heart’s right atrium to allow perfusion with Ringer’s solution for 2 min. When the liver’s color started turning from red to pale red, 4% paraformaldehyde (PFA) was perfused for fixation for 10 min. After the perfusion, the brain was collected and post-fixed in 4% PFA overnight at 4 °C. The following day, the brain was washed and stored in PBS at 4 °C. The brain was sectioned into 50 μm coronal sections using a Vibratome (Leica VT1000S) and stored in PBS, protected from light until mounting on a microscope slide with Vectashield Antifade Mounting Medium (H-1000).

#### Retina sample preparation

The mice were killed by cervical dislocation at ZT5-6, then both eyes were taken out, and their cornea and lens were removed. The eye cup was fixed in 4% PFA at room temperature for two hours, washed in PBS, and stored at 4 °C, protected from light. The retina was then either removed from the eyecup and directly mounted or prepared for cryosection (see below). Four radial incisions were made to flatten out the sample on a microscope slide and mounted using Vectashield Antifade Mounting Medium (H-1000). For cryosections, the eyecup was incubated overnight in 15% then 30% sucrose solution at 4 °C. The eyecup was then embedded in Tissue-Tek OCT compound (Sakura Finetek USA, Torrance, CA), frozen on dry ice and stored at −80 °C until use. The eyecup was then sectioned with a cryostat onto 20 μm thick sections.

#### Immunostaining

The retina was permeabilized with 0.3% Triton X-100 for one hour at room temperature then saturated with blocking solution (5% Horse Normal Serum) for two nights at 4 °C. The retina was first incubated with a primary antibody (F006 anti-mouse melanopsin polyclonal antibody; Advanced Targeting Systems, San Diego, CA) for two days at 4 °C, then with the secondary antibody (Donkey anti-Rabbit Cy3, 711-165-152, Jackson) for four hours at room temperature and washed with PBS after each step. The retinas were then flattened on a glass microscopy slide and mounted with Vectashield Antifade Mounting Medium (H-1000). The whole staining process was done in a 48-well plate protected from light, under constant agitation.

#### Confocal microscopy imaging

The brain and retina samples were imaged on a Zeiss LSM 880 confocal microscope. An overview of the tissue was first done at 10X magnification to determine the regions of interest to image. These regions were then imaged at 20X magnification with 10% overlap between adjacent fields of view and interval of 1.5 μm on z-axis. Raw images and maximum intensity projection obtained on Zen software (Zeiss) were then exported.

#### Cell quantification

The quantification of OPN4 and OPN5 cells in the retina was done using Qupath software^35^ on the maximum intensity projection images. Cells were first automatically detected using the “cell detection” function with parameters optimized on a small area of the retina. Then, the whole retina was manually scanned to eliminate false positive detection. The area of the detected cells was then calculated in Qupath and extracted.

#### Tissue preparation for SBEM imaging

Tissue was prepared for SBEM as previously described^[38]^. Before the volume was collected, a low magnification (∼500×) image was collected of the block face to confirm the anatomical location of the volume based on tissue landmarks. The region selected for SBEM was in the central region of the retina. The retina volume was collected in 2.0 to 2.4 kV accelerating voltages, with a raster size of 20k×20k and pixel dwell time of 0.5–1.5 µs. Once the volume was collected, the histograms for the slices throughout the volume stack were normalized to correct for drift in image intensity during acquisition. Digital micrograph files were normalized using Digital Micrograph and then converted to MRC format. The stacks were converted to 8-bit, mosaics were stitched. The final image volume comprises 948 sections, each measuring 50 nm in thickness. Each section consists of an array of 20,000 by 20,000 pixels, achieving a high resolution of 5.61 pixels per nanometer.

### Semi-automatic segmentation

We automatically detected and traced dendrites marked with APEX2 that appear dark in EM images using the *imodauto* function of the software IMOD[^36^]. The function used image thresholding to segment objects. Here, we detected all pixel values under a fixed threshold corresponding to the average value of the labeled dendrites. If the pixels detected formed a large enough area, they are saved as a contour. Once all images of the volume are processed, we meshed all contours to create 3D surface with contours overlapping over multiple sections (*imodmesh*). The surfaces were then separated by different objects (*imodsortsurf*). We then used a custom Python script to delete all objects that were smaller than 15 sections to eliminate small structures such as lysosomes that also appear dark in EM images. Lastly, all remaining objects in every section were manually checked to confirm that they only contained labeled dendrites.

### Manual segmentation

#### 3D reconstruction of neuropsin expressing cells

VAST Lite software^21^ was used for proofreading and manual segmentation. MRC image volume was extracted as individual TIFF images and then imported into VAST using in-software tools. Each cell was fully traced as a separate object by drawing the cell body and dendrites as it moved through each slice of the volume. The same process was used to trace organelles (mitochondria, nucleus) in the same cells. Individual volumes and 3D objects were extracted using the VAST Tools script in Matlab and were visualized with the software Blender.

#### Neuropsin RGCs dendrite coverage

We divided the volume into a 5 × 5 grid and randomly selected five boxes, excluding the ones containing the cell body of a neuropsin-expressing RGC. We then traced all labeled dendrites to estimate the percentage of the retina surface covered by neuropsin cell dendrites.

#### Training and quality control

All person involved in manual tracing were first trained to identify and label specific structures (axons, dendrites, soma) and ultrastructures (mitochondria, synapses, etc.) in non-labelled cells, then worked on proofreading and completing the automatic segmentation. All data were systematically re-checked at least once by a different person than the one who initially produced it.

### Electrophysiology

#### Multielectrode array recording

Retina recordings were done as described previously^37^. The retinas of OPN5^Cre/Cre^, OPN4^Cre/Cre^ or WT littermates were mounted on 256-electrodes MEA (Multichannel Systems, Reutlingen, Germany) with ganglion cells facing down and continuously perfused with oxygenated cells artificial cerebrospinal fluid (aCSF) at 34 °C. The synaptic blockers cocktail used contains D-AP5 (50µM), CNQX (20µM), and L-AP4 (50µM). The chromophore supply consists of the addition to aCSF of 11-cis-retinal dissolved in 0.1% ethanol. Negative thresholds for spike detection were set at 5 times the standard deviation (SD) of the noise on each channel. Spike cut-outs, consisting of 1 ms preceding and 2 ms after a suprathreshold event, along with a timestamp of the trigger were written to the hard disk. Two retina patches from three different animals were recorded in each corresponding experiment.

#### Light stimulation

Recorded retinas were exposed to full field light stimulation of increasing irradiance (5.10^11^ photons/cm^2^/s to 1.10^15^ photons/cm^2^/s) using monochromatic LEDs (LuxeonStar 5, luxeonstar.com). Irradiances and durations of the light stimulations were controlled by custom Python scripts.

#### Analysis

For each channel, spike cut-outs were sorted into trains of a single cell using Offline Sorter (Plexon, Denton, TX). Data analysis and display were performed using Neuroexplorer (Plexon) and custom Matlab scripts (Mathworks, Natick, MA). Different parameters of the response of the cell to light were determined. The response discharge rate was calculated as the average of the action potential discharge during the response duration, minus the average discharge rate 30 s before the start of the light stimulation (baseline). The duration of the response was defined as the time while the discharge rate was over a threshold defined as the baseline + 2*SD. The response was considered over when the discharge rate was below the threshold for 3 consecutive seconds. To consider that a cell responds to light stimulation, the duration of its response should last longer than 1s.

### Statistical analysis

Results were expressed as means ± standard error of the mean (SEM). Comparisons of the two groups were done using a non-parametric rank-sum test (Mann-Whitney U Test). Comparisons of multiple groups were done by using ANOVA on ranks (Kruskal-Wallis H test) followed by a post hoc test (Pairwise Mann-Whitney U-tests) with Benjamini-Hochberg p-value correction. A statistically significant difference was assumed with a corrected p-value inferior to 0.05. All statistical analyses were performed using R (R project). All custom scripts generated in this study are available on GitHub (https://github.com/Carboneinerte).

## Supplementary Information

Below is the link to the electronic supplementary material.

Supplementary Material 1

Supplementary Material 2

## Data Availability

No datasets were generated or analysed during the current study.
