# Supplementary figures and images for: Neuropsin-expressing cells in the retina affect melanopsin expression and response to light in mice
Source: Sci Rep. 2026 Jan 8;16:1082. doi: 10.1038/s41598-025-22815-4 (PMC12783770; doi:10.1038/s41598-025-22815-4)

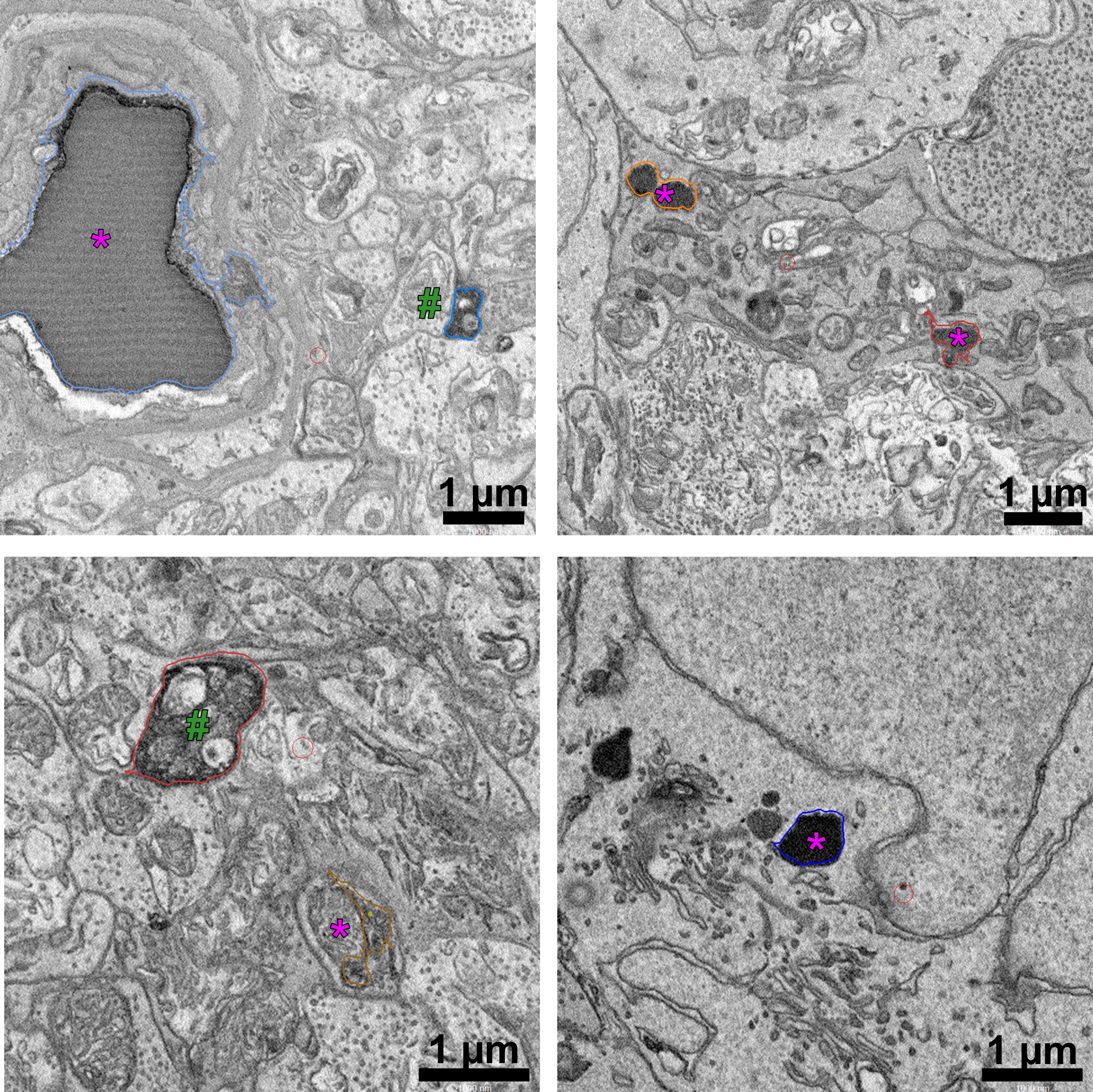

Supplement: Supplementary file 2 — Supplementary Material 2 [file 41598_2025_22815_MOESM2_ESM.png]
